# Personality and morphological traits affect pigeon survival from raptor attacks

**DOI:** 10.1038/srep15490

**Published:** 2015-10-22

**Authors:** Carlos D. Santos, Julia F. Cramer, Liviu G. Pârâu, Ana C. Miranda, Martin Wikelski, Dina K. N. Dechmann

**Affiliations:** 1 Department of Migration and Immuno-ecology, Max Planck Institute for Ornithology, Am Obstberg 1, 78315 Radolfzell, Germany; 2 Departamento de Biologia, Centro de Ciências Biológicas e da Saúde, Universidade Federal do Maranhão, Campus do Bacanga, 65080-040, São Luís, MA, Brazil; 3 Department of Biology, University of Konstanz, 78464 Konstanz, Germany

## Abstract

Personality traits have recently been shown to impact fitness in different animal species, potentially making them similarly relevant drivers as morphological and life history traits along the evolutionary pathways of organisms. Predation is a major force of natural selection through its deterministic effects on individual survival, but how predation pressure has helped to shape personality trait selection, especially in free-ranging animals, remains poorly understood. We used high-precision GPS tracking to follow whole flocks of homing pigeons (*Columba livia*) with known personalities and morphology during homing flights where they were severely predated by raptors. This allowed us to determine how the personality and morphology traits of pigeons may affect their risk of being predated by raptors. Our survival model showed that individual pigeons, which were more tolerant to human approach, slower to escape from a confined environment, more resistant to human handling, with larger tarsi, and with lighter plumage, were more likely to be predated by raptors. We provide rare empirical evidence that the personality of prey influences their risk of being predated under free-ranging circumstances.

Evolutionary biologists have recently drawn attention to individually consistent animal behaviours as potential targets of natural selection^1^. Individual behavioural variation has been classically treated as non-adaptive noise of potentially adaptive population averages^2^. However, there is now growing evidence that individual variation in behaviour is often distributed in a non-random manner, reflecting systematic responses of individuals to their surrounding environment^3^. These consistent behaviours are commonly named personality traits, coping styles, or temperaments^2^, and have been documented in more than 100 species across various taxonomic groups^4^. Personality traits were found to have a genetic component in a number of cases^5^ and to impact individual fitness (individual survival and reproductive success^6^), thus potentially driving evolutionary pathways of organisms.

Predation is a major force of natural selection. Predators often have marked preferences for certain traits of their prey, thus shaping trait frequency in the prey population^7^. The variability of individual prey behaviour have long been treated as non-adaptive, in contrast with that of morphological and life history traits^2^. However, recent studies have shown that prey do exhibit individual consistency regarding predator avoidance behaviour across time and contexts, thus creating the potential for differential selection driven by predation^8^,^9^,^10^. Several studies have found, for example, that exploratory behaviour of prey under predation threat is highly consistent within individuals and variable across individuals^11^,^12^,^13^. Similar findings were described for other predator avoidance behaviours. Individuals were found to respond consistently to the presence of a predator in their levels of vigilance^14^, escape strategies^9^,^15^,^16^, and even aggression towards the predator^9^,^10^. Nevertheless, the evidence that these personality traits affect prey survival is still very scarce (but see^6^), which greatly limits our current understanding of the actual role of personality in the evolutionary pathways of prey populations.

Group-living species are usually considered at the level of the group rather than at the level of the individual^17^. However, groups provide a diversity of individual traits that are potentially subjected to non-random predator selection^17^. Targeting prey with unusual morphology or behaviour can help predators to focus on a single individual, alleviating the confusion effect of the evading group^18^,^19^. Phenotypic assortment by predators over grouping prey has been frequently demonstrated for morphological and life history traits, including coloration^19^,^20^,^21^, body size^7^,^22^, body shape^23^, body condition^24^, sex^22^,^25^ and age^22^,^26^,^27^,^28^. Far fewer studies have established a link between personality traits of grouping prey and their survival from predators^27^,^29^,^30^,^31^,^32^,^33^,^34^,^35^. Furthermore, most of these studies were conducted with fish in laboratory-controlled conditions, which limits the generalization of their results, particularly regarding their relevance for animals in their natural environments. The lack of evidence from wild predator-prey systems is likely due to the logistical challenge of monitoring predation events, but recent innovations in animal GPS tracking now allow to overcome these difficulties. In fact, animals with known personalities can now be tracked in the wild, where they are confronted by natural predators, and their fates can be carefully documented.

In this study we GPS-tracked whole flocks of pigeons (*Columba livia*) while homing over areas populated by raptor predators. Tracking data provided evidence for raptor attacks on the flying pigeons and this was complemented by field surveys at the attack sites. Prior to homing flights, predator avoidance behaviour of individual pigeons was tested in four personality challenges, namely tolerance to human approach, resistance to human handling, ability to leave a confined environment, and the avoidance of novel objects. The first three challenges are expected to reflect survival strategies of pigeons when attacked by a raptor, i.e. when pigeons are chased by the raptor in an open space, when pigeons are handled by the raptor after a non-lethal attack, and when pigeons are chased by the raptor in a confined space (e.g. forest patch). The fourth challenge is commonly used to measure risk taking behaviour^36^. In addition, we quantified six morphological variables in all our pigeons, which may be relevant for predators, namely, body weight, wing length, tail length, tarsus length, pectoral circumference and plumage coloration. Morphological and personality variables were included as predictors in survival models aiming to explain predation rates of homing pigeons by raptors. We hypothesized personality traits to be as relevant as morphological traits for pigeon survivorship during raptor attacks. Our study is one of only few testing the influence of prey personality on their survival from predators and to our best knowledge the only one using tracking technology to do so in free-ranging animals.

## Results

Fifteen out of our 27 homing pigeons were predated throughout the course of this study (10 males and five females). Homing trips lasted between 15 minutes to an extreme of 2 days, when flocks experienced raptor attacks. Pigeons attacked by raptors often stopped for long periods and/or made long detours on their way home to avoid the areas where attacks occurred. For the seven cases where the predation occurred during high-resolution tracking we were able to confirm that the predated birds were flying in a flock (see Supplementary Fig. S1a for an example).

In the behavioural challenges (flight initiation distance (FID), escape reaction time (ERT), tonic immobility (TI) and feeder neophobia (FN), see Methods for details), birds responded in a highly consistent manner across repeated trials (Repeatability = 0.44, 0.31, 0.45 and 0.51, for FID, ERT, TI and NF respectively; P < 0.001 in all cases).

**Figure 1:**
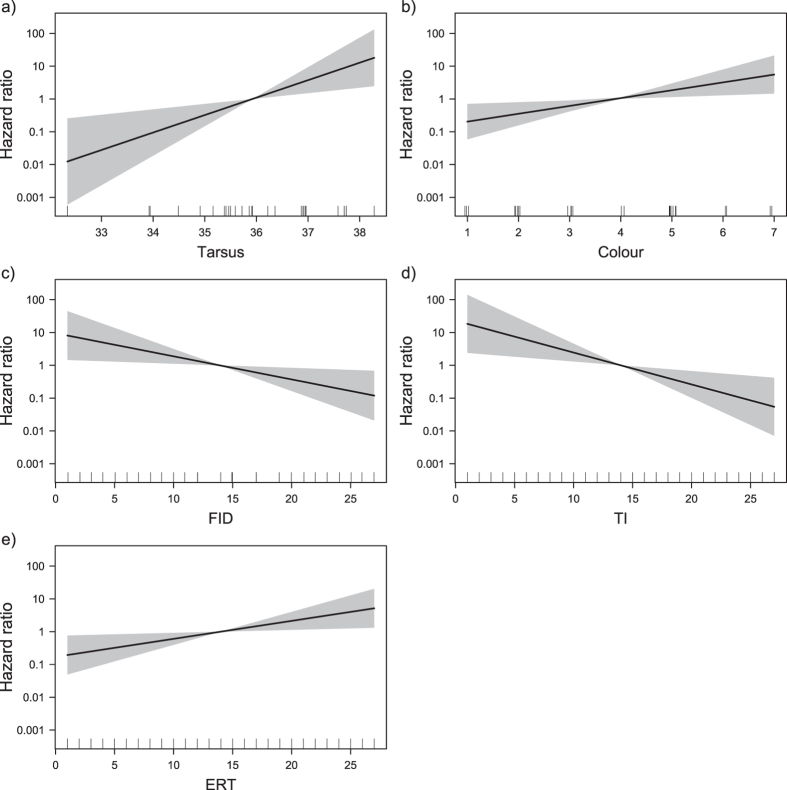
(**a**–**e**) Predicted effects of morphological and personality traits on the hazard ratio based on the reduced Cox proportional hazard model. Only covariates with significant effect are shown. Shading represents 95% confidence intervals. Rugs in (**b**) and (**c**) were jittered for clarity. Tarsus length (**a**) is in mm. Increasing colour values represent lighter animals. FID - flight initiation distance; TI - tonic immobility; ERT - escape reaction time.

The Cox proportional hazard reduced model shows that both morphological and personality traits are important to explain pigeon survival (Table 1). Tarsus length and plumage lightness were the morphological predictors with relevant effect on pigeon survival (Table 1 and 2). Birds with longer tarsi and lighter plumage were more likely to get predated by raptors. Each additional millimetre in tarsus length tripled the hazard rate (exp(β) = 3.41, Fig. 1a). Plumage lightness increased hazard rate by 73% per additional score in the colour index (exp(β) = 1.73, Fig. 1b). From the personality predictors three, out of four, had significant effects on survival of the pigeons, namely FID, TI and ERT (Table 2). All these predictors had similar impacts on hazard rates, with 13 to 20% of change per unit of behavioural score (exp(β) = 0.85, 0.80 and 1.13, for FID, TI and ERT). Both FID and TI had a negative effect on the hazard rate (Fig. 1c,d). Birds with longer FIDs (i.e. less tolerant of a human approach) and less resistant to immobilization (i.e. longer TIs) were less likely to be predated. Finally, ERT had a positive effect on the hazard rate, i.e. birds that took longer to escape from an enclosure were more likely to be predated (Fig. 1e).

Plumage oddity did not explain the influence of plumage coloration on hazard rates better than plumage lightness, and the inclusion of rump coloration violated the proportionality assumption of the model, thus we could not test the effect of this predictor (Supplementary Table S1).

## Discussion

Our results show that homing pigeons exhibited consistent responses to standardized experimental predator avoidance challenges, these being considered traits of their personality. Importantly, we also show that pigeon personality traits translated into survival probabilities during predatory attacks by raptors predator attacks. Specifically, pigeons that escaped from an approaching human at longer distances (FID) were less likely to be killed by raptors during homing trips. Similarly, birds that escaped from a confined environment more quickly (ERT) were more likely to survive. Finally, birds that were more resistant to human handling (TI) were more likely to be killed by raptors. The usefulness of all these anti-predator behaviours can easily be applied to circumstances in the wild. Low tolerance to initial predator approach could give birds a distance advantage over the chasing raptor, thus increasing the chances of escaping^37^. Birds chased by a raptor may search for refuge in forest patches or scrubs (which was often observed from the tracking data), where they might end up entrapped by the predator in this confined environment. Therefore, the ability to quickly escape from confined spaces may increase chances of survival. Finally, for birds captured by a raptor but not immediately killed, “playing possum” and waiting for an escape opportunity may offer an important advantage^38^. On the contrary, birds more prone to struggle while immobilized likely have more chances of triggering a fatal strike of the predator. In fact, other pigeons of the same breeder frequently return injured to their lofts, indicating that non-lethal attacks of raptors often occur (Supplementary Fig. S1c). This suggests that tonic immobility might be a very effective anti-predator strategy for homing pigeons in the study area. Morphological traits also played an important role for the survival of pigeons from raptor attacks. Specifically, plumage coloration and tarsus length were significantly linked with rates of predation. Lighter coloured pigeons were more likely to be predated than their darker flock mates. A previous study showed that goshawks (*Accipiter gentilis*) preyed preferentially upon white feral pigeons despite the fact that they were rare in the population^19^. However, in this study plumage oddity rather than lightness was the key factor explaining the prey selection by the goshawks. This is supported by another study showing that goshawks switched their preferences from white to dark pigeons when the frequency of dark pigeons was manipulated to become the rare phenotype^39^. We tested the effect of plumage oddity in alternative to plumage lightness and did not confirm the conclusions of these two studies. While plumage oddity had no effect on the survival of our pigeons, plumage lightness showed a significant effect (Supplementary Table S1). We argue that plumage lightness should dramatically increase distinctiveness of pigeons in the mainly dark green landscape of the study area, diminishing the confusion effect for the chasing predator and explaining its preference. Another study found that peregrine falcons (*Falco peregrinus*) avoid pigeons of the “wild” variant plumage due to the presence of the white contrasting rump^20^. The rump is suggested to generate confusion for the predator given that pigeons alternatively expose the contrasting white rump, and grey wings and body during the evasive manoeuvring. However, the authors also acknowledge that a white rump could prove disadvantageous when the pigeons seek cover among trees, which was frequently the case in our study. Despite the interest of this topic, we could not evaluate the effect of the white rump in our study as its inclusion as a variable in our model failed to meet modelling assumptions. Finally, our model also showed a strong effect of tarsus length on the survival of pigeons. Pigeons with longer tarsi were more prone to predation. Tarsus length is a proxy of body size, which greatly varies in domestic pigeons and likely influences their escape performance. In fact, smaller pigeons should have higher manoeuvrability and escape speed than larger pigeons, which has been verified in other bird species^40^,^41^.

We should note that the raptor predators probably responsible for the attacks, the peregrine falcon and the goshawk, could have contributed differently to the observed results, particularly regarding the personality traits. In fact, longer FID could be more useful during attacks by a goshawk that shows relatively slow chasing speeds. But escape reaction distance should be less relevant when confronted with the exceptional speed of the peregrine falcon^42^. In contrast, the speed at which to leave a confined space should be more important to pigeons attacked by the peregrine falcon, which is typically an open space predator^42^, while the goshawk is a forest specialist and thus less likely to let a pigeon escape in such an environment^42^. It should be emphasized that we kept our pigeons in the loft except when we subjected them to homing releases or training around the loft. Thus, their flight performance in concealed forest environments is expected to be very limited when compared with that of their wild predators.

Our study provides rare empirical evidence that personality and morphological traits of prey can simultaneously influence prey survival from predators (also see refs 30,33,34.). This opens up the possibility for multiple interactions of predation pressures that may cancel each other and contribute to the maintenance of a diversity of phenotypes in the population, something that has been highly debated in recent personality literature^35^,^43^,^44^,^45^,^46^. Our study is also uncommon in presenting evidence of predation on grouping prey. Although, we could only directly document raptor attacks on the pigeon flocks in a subset of our data, we have no reason to doubt that this phenomenon extended to the remaining events of predation. Therefore, we are convinced that our results are explained by phenotypic selection of specific personality and morphological traits of the pigeons available in our flocks by the raptors. This study also illustrates how modern tracking methods allow us to take the study of prey-predator interactions out of the lab and into the wild. Tracking allowed us to determine the causes of death of our homing pigeons, and in several cases to document precisely how and when predators attacked our flocks (Supplementary Table S2 and Fig. S1a). The relevance of personality traits for prey survival remains poorly known in free-ranging animals but we hope that this study encourages the use of tracking methods to overcome logistic difficulties in studying this exciting topic.

## Methods

### Ethical note

The experimental procedures of this study were performed in accordance with the German regulation on animal experimentation. License number 35–9185.81/G-13/19 approved by the Ethical Committee of Baden-Württemberg (Regierungspräsidium Freiburg Abteilung 3).

### Subjects and study site

This study was conducted with 27 one-year-old homing pigeons, 17 males and 10 females. Pigeons belonged to a breeder and were housed near Eigeltingen (47°53′37′′N, 8°55′35′′E), Southern Germany. Males and females were kept in separate home lofts during the course of behavioural tests and homing flights to prevent breeding, which could affect the results of our experiments. All pigeons had participated in the same races during the previous year, with homing distances ranging from 140 to 240 km, thus they all had similar levels of flight experience. Before the experimental homing flights, we measured weight, wing length, tail length, tarsus length, and pectoral circumference in all the individuals. In addition, an index of plumage lightness was built from pictures of each pigeon upperparts with the wings open (named “colour” in the results). Plumage was ranked from uniform dark blue (scored as 1) to uniform white (scored as 7), with the blue-grey “wild” variant at the centre of the distribution (scored as 4). The landscape surrounding the home lofts, where we conducted homing flights, comprises a mosaic of forest patches, pastures and cultivated fields. Peregrine falcons and goshawks are recognized by local breeders as the main predators of homing pigeons in the area, and likely responsible for losses of up to 50% in each racing season. Atlas surveys identified five pairs of peregrine falcon and eight pairs of goshawk in the study area^47^.

### Behavioural challenges

We subjected the experimental pigeons to four different behavioural challenges (flight initiation distance (FID), escape reaction time (ERT), tonic immobility (TI) and feeder neophobia (FN)) aiming to phenotype personality traits in multiple dimensions of predator avoidance behaviour. Subjects were tested individually in three repeated trials of each challenge to determine the consistency of their behaviour. Challenges were conducted from November 2012 to February 2013, alternatingly and with three-week breaks between trials of each challenge. Individuals were tested in a randomized sequence between trials.

FID evaluated the tolerance of birds to the approach of an unfamiliar person (different unfamiliar person in different trials). Each pigeon was placed under a cardboard box on a high table (1.5 m) with a restricted surface (40 × 40 cm). After an acclimatization period of 1 minute, the box was lifted with a string-pulley-system and the human chaser moved towards the bird from a distance of 2.5 m with constant slow speed until the pigeon took flight. The distance between the chaser’s chest and the bird at the moment of take-off was measured as the variable of interest.

In the ERT challenges, we measured the ability of birds to escape from a confined space, which was assumed to be a threatening circumstance. Birds were placed individually in a closed cardboard box (50 × 40 × 40 cm) and moved a hundred meters from the home loft to where no landmarks were visible from within the box when the top was open. After 1 minute of acclimatization, the top of the box was opened with a string in a way that the pigeon could not see the observer, and the time taken by the pigeon to exit the box was measured.

TI was induced by placing each individual on its back on a table while exerting slight pressure to the breast, simulating the immobilization caused by a predator attack. The bird was released after 12 seconds and the time until it recovered from paralysis was recorded as variable of interest. A different person than the human chasers of FID conducted this experiment.

Finally, in the FN challenges the pigeons’ familiar feeder was visually modified with unfamiliar objects and offered to the birds with abundant food after 1 day of food deprivation. A different set of objects (shiny ribbons, colourful tubes and small stuffed toys) was used in each of the three trials to avoid habituation. Birds were individually placed under a cardboard box in their home loft and exposed to the manipulated feeder by lifting the box with a string-pulley-system after 1 minute of acclimatization. Neophobia was measured as the time taken by the birds to start feeding. All birds started feeding within 9 seconds to 9 minutes. An experimental control, where the feeder was offered to the birds without manipulation, was conducted in the day before each trial. This allowed controlling for individual differences in feeding motivation regardless the feeder manipulation.

### Homing flights and predation surveys

We released each of the two experimental groups, female-group (10 birds) and male-group (17 birds), in five homing flights from April to May 2013. Releases of each group were separated in time from 4 days to 2 weeks. Flocks were released from increasing distances to the home loft (2 to 15 km away) in separate directions for males and females (southwest and southeast, respectively). In eight, out of the 10 flights, pigeons were tracked with miniaturized GPS-GSM data loggers (20 g, TT-202/R9C5 module, Think Technology, Belgium). Data loggers delivered GPS positions remotely (through the GSM network) every 2 seconds for 5 hours after a release and every 4 hours over the following week. This allowed the detailed reconstruction of homing trips and the detection of predation events on those pigeons that did not return (see Supplementary Fig. S1a for an example). Radio transmitters (0.8 g, Holohil Systems Ltd., Canada) were glued to the GPS loggers in order to facilitate their recovery when dropped in areas covered by vegetation. Logger backpacks were protected from rain with a layer of duct tape and attached to the pigeons by a small Velcro strip glued to the dorsal feathers which had been clipped down to 1 cm. Subjects carried 20 g dummy loggers in prior to data collection in order to adapt their flying to the extra load. In the two untracked flights, pigeons carried dummies and were released from the shortest homing distance (2 km).

We ground-truthed attack sites for evidence of predation when pigeons did not home within the release day and if either GPS fixes were reported from the same spot over several hours or if there was a sudden failure of the logger while the battery level was still high. Logger failure never occurred in pigeons that homed successfully, and most loggers we recovered after predation events showed battery perforation and/or damage of electronic components. We should emphasize that loggers were only protected by a layer of duct tape and the lithium-ion polymer batteries were wrapped in a soft package with laminated film dividing the chambers of the electrolyte. Therefore, chances of battery damage by predators were very high. During field surveys for the causes of predation, freshly plucked feathers, indicative of raptor predation, were often found near the last GPS position. In contrast, we never found feathers cut by teeth, or loggers and attachment skin patches (Supplementary Fig. S1b) with teeth marks, as would be typical for mammalian predation. Tracking data were also carefully inspected for evidence of raptor predation. Evidence included flock dispersal instants before the lost bird stopped moving (illustrated in Supplementary Fig. S1a); sudden drop of altitude to ground level by the lost bird, instants before it stopped moving; or logger failure during active flight. Field and tracking evidence for raptor predation are presented for each case in Supplementary Table S2. We included the four birds that were lost during untracked flight in our analysis as predated by raptors based on the strong evidence of raptor predation for all losses in the tracked flights (Supplementary Table S2 and Fig. S1). It should be noted that the 2 km release distance in the untracked releases were within the familiar training zone of these pigeons. Thus it is highly unlikely that causes other than predation explain the disappearance of these pigeons. In addition, attacks by goshawks and peregrine falcons on training pigeons of the breeder were frequently observed in the area surrounding the loft during the time our study took place.

### Data analysis

We used Cox proportional hazards modelling^48^ to test for the effects of personality and morphological traits on the survivorship of our pigeons during predator attacks. The Cox proportional hazards model is a multiple regression technique that estimates the hazard ratio while allowing for the simultaneous adjustment of influential covariates on survival time. Hazard rates of covariates can be calculated from the model coefficients (exp(β)) as the percentage of change in mortality risk per unit of the covariate (increase of mortality risk if exp(β) > 1 or decrease of mortality risk if exp(β) < 1). For instance, a covariate with exp(β) = 1.05 contributes to the increase of the mortality risk by 5% per unit, but if exp(β) = 0.95 the covariate contributes to the decrease the mortality risk by 5% per unit^49^. The behavioural responses of birds in different trials of behavioural challenges were checked for repeatability, as a condition for them to be interpreted as personality traits. We used “adjusted repeatability” estimation following Nakagawa and Schielzeth^50^ in order to control for potential confounding effects (set as random effects) of the trial, in all challenges, and the feeding motivation in the NF challenge. P-values of adjusted repeatabilities were generated by randomization (1000 permutations) as recommended by Nakagawa and Schielzeth^50^. The normality of model residuals was verified in all cases. In order to be used as predictors in the survival models, behavioural responses of individuals in different trials were averaged and the resulting variables were rank transformed to prevent the over-influence of extreme values on model outputs. Tail length was not included as predictor in the models due to a high correlation with wing length (Pearson’s correlation = 0.83). All the remaining traits were not correlated enough to add redundancy to our models, thus they were all used as predictors (Supplementary Table S3). The response variable in survival models combined flight sequence number (as a time-dependent variable) and the occurrence of mortality events due to predation. The resulting variable was right-censored, because some pigeons were still alive at the end of the experiment. Sex was included as stratification variable in models since males and females formed different experimental groups and flew in different flights, and therefore conclusions cannot be drawn from our study as to the influence of sex on survivorship. Assumptions of proportional hazards and no interaction suggested for stratified Cox models were checked following Kleinbaum and Klein^49^. The full model was reduced by AIC-based backward stepwise elimination and conclusions were drawn from predictors with significant effect on pigeon survival in the reduced model. We also built models where plumage lightness was replaced by either plumage oddity or rump coloration, since these variables were shown before to influence predation rates of pigeons by raptors^20^,^39^. Both of these variables were dichotomous, presence vs absence of the white rump, and “wild” blue-grey plumage variant vs others (odd plumages).

All computations were done with the free statistical software R^51^. Repeatability analysis was carried out with the rpt.remlLMM.adj function from the rptR package^52^, and the survival analysis was conducted with the coxph function from the survival package^53^.

## Additional Information

**How to cite this article**: Santos, C. D. *et al.* Personality and morphological traits affect pigeon survival from raptor attacks. *Sci. Rep.* **5**, 15490; doi: 10.1038/srep15490 (2015).

## Supplementary Material

Supplementary Information

## Figures and Tables

**Table 1:** Cox proportional hazard model comparison by the Akaike’s information criterion (AIC).

Model	Parameters	Log-likelihood	AIC	ΔAIC	ω
Full	Tarsus, Wing, Weight, Colour, Pectoral, FID, TI, ERT, FN	−23.98	65.97	2.96	0.19
Reduced	Tarsus, Colour, FID, TI, ERT, FN	–25.50	63.01	0	0.81

The full model includes all predictors and the reduced model includes predictors selected from the full model by AIC-based backward stepwise elimination. Log-likelihood values, AIC, difference from lowest AIC score (ΔAIC) and Akaike weights (ω) are shown for each model. FID - flight initiation distance; TI - tonic immobility; ERT - escape reaction time; FN - feeder neophobia.

**Table 2:** Estimated parameters of Cox proportional hazards reduced model.

Covariate	β	exp(β)	SE	*Z*	95% CI	P
Tarsus	1.226	3.407	0.423	2.90	1.488, 7.800	0.004
Colour	0.551	1.734	0.215	2.56	1.138, 2.642	0.010
FID	−0.162	0.850	0.067	−2.44	0.746, 0.969	0.015
TI	−0.224	0.799	0.078	−2.86	0.686, 0.932	0.004
ERT	0.126	1.134	0.053	2.40	1.023, 1.258	0.016
FN	0.100	1.105	0.053	1.90	0.997, 1.225	0.057

Coefficient estimates (β), exponent of coefficients (exp(β)), standard errors (SE), *Z* statistics, 95% confidence intervals (95% CI), and P-value (P) are presented. FID - flight initiation distance; TI - tonic immobility; ERT - escape reaction time; FN - feeder neophobia.
