# Supplementary material for: Personality and morphological traits affect pigeon survival from raptor attacks
Source: Sci Rep. 2015 Oct 22;5:15490. doi: 10.1038/srep15490 (PMC4614540; doi:10.1038/srep15490)

Santos, C.D., Cramer, J.F, Pârâu, L.G., Miranda, A.C., Wikelski, M. & Dechmann, D.K.N. Personality and morphological traits affect pigeon survival from raptor attacks.

Supplementary Figure S1. Evidence for pigeon predation by raptors. (a) Tracking data showing a predation event. Pigeons were moving SW as a flock when attacked. Remains of the predated pigeon (white track) and its GPS logger were found at the head of its track (position marked by +). Remaining animals of the flock (red tracks) temporarily dispersed after the predation event. Forest patches and pastures are represented in dark green and light green, respectively. (b) GPS logger with attachment skin patch recovered from a predated pigeon. (c) Injured pigeon that escaped from a raptor attack. The background map in (a) was created with the free GIS software QGIS, using satellite images publically available at the Earth Science Data Interface (ESDI).

Supplementary Table S1. Estimated parameters for Cox proportional hazard models with alternative variables for plumage coloration (plumage lightness and plumage oddity). Coefficient estimates (β), exponent of coefficients (exp(β)), standard errors (SE), *Z* statistics, 95% confidence intervals (95% CI), and P-value (P) are shown for each model. FID - flight initiation distance; TI - tonic immobility; ERT - escape reaction time; FN - feeder neophobia.

Covariate	β	exp(β)	SE	*Z*	95% CI	P
Full model with plumage lightness (Colour)						
Tarsus	1.445	4.240	0.636	2.27	1.218, 14.760	0.023
Wing	0.016	1.017	0.011	1.51	0.995, 1.038	0.130
Weight	0.001	1.001	0.019	0.05	0.964, 1.039	0.959
**Colour**	**0.529**	**1.697**	**0.259**	**2.04**	**1.021, 2.821**	**0.041**
Pectoral	0.005	1.005	0.004	1.18	0.997, 1.012	0.240
FID	-0.252	0.777	0.107	-2.36	0.630, 0.959	0.019
TI	-0.310	0.733	0.116	-2.68	0.585, 0.920	0.007
ERT	0.116	1.124	0.053	2.19	1.012, 1.247	0.029
FN	0.123	1.131	0.067	1.84	0.992, 1.290	0.066
Full model with plumage oddity (Odd)						
Tarsus	1.308	3.700	0.604	2.17	1.134, 12.077	0.030
Wing	0.016	1.017	0.010	1.60	0.996, 1.037	0.110
Weight	0.004	1.004	0.018	0.21	0.969, 1.039	0.838
**Odd**	**1.067**	**2.907**	**0.791**	**1.35**	**0.617, 13.699**	**0.177**
Pectoral	0.007	1.007	0.004	1.89	1.000, 1.014	0.058
FID	-0.192	0.825	0.085	-2.25	0.698, 0.975	0.024

TI	-0.236	0.790	0.093	-2.53	0.658, 0.948	0.011
ERT	0.087	1.091	0.045	1.93	0.999, 1.193	0.054

Supplementary Table S2. Field and tracking evidence for pigeon losses due to raptor predation.

Pigeon id	Flight where lost (M – Males; F – Females)	Field evidence				Tracking evidence				Losses during non-tracked flights
						Losses during continuous tracking (positions every 2 seconds)			Losses during discrete tracking (positions every 4 hours)	
		Logger found in a peregrine falcon nest	Freshly plucked feathers found	Logger and/or attachment skin patch found without mammalian teeth markings	Feathers, logger and/or skin patch found near a peregrine falcon nest	Bird dropped from higher than 50 meters to ground level near the last GPS position	Logger stopped in flight with high battery charge (i.e. battery or logger damage)	Flock dispersion coinciding with the loss of a bird	Logger stopped with high battery charge (i.e. battery or logger damage)	
P1	3 M		X			X		X		

P3	1 M									X
P4	3 M			X		X				
P5	5 M		X		X	X		X		
P7	1 M									X
P8	4 M	X	X	X	X			X		
P14	5 M								X	
P15	5 M			X	X			X		
P16	1 M						X			
P17	5 M								X	
P21	4 F								X	
P22	1 F									X
P23	1 F									X
P26	2 F			X					X	
P27	5 F		X	X		X				

Supplementary Table S3. Spearman’s correlation matrix for the predictors used in the Cox proportional hazard models.

	Tarsus	Wing	Weight	Colour	Pectoral	FID	TI	ERT	FN
Tarsus	1	0.46	0.58	0.12	0.21	-0.11	0.29	-0.25	0.05
Wing	0.46	1	0.27	0.10	-0.11	-0.14	0.28	0.04	0.08
Weight	0.58	0.27	1	0.25	0.46	-0.30	0.60	0.11	0.44
Colour	0.12	0.10	0.25	1	0.53	0.11	0.23	-0.07	0.09
Pectoral	0.21	-0.11	0.46	0.53	1	-0.03	0.39	0.12	0.27
FID	-0.11	-0.14	-0.30	0.11	-0.03	1	-0.55	-0.25	-0.02
TI	0.29	0.28	0.60	0.23	0.39	-0.55	1	0.17	0.42
ERT	-0.25	0.04	0.11	-0.07	0.12	-0.25	0.17	1	0.22
FN	0.05	0.08	0.44	0.09	0.27	-0.02	0.42	0.22	1

Supplementary Figure S1.

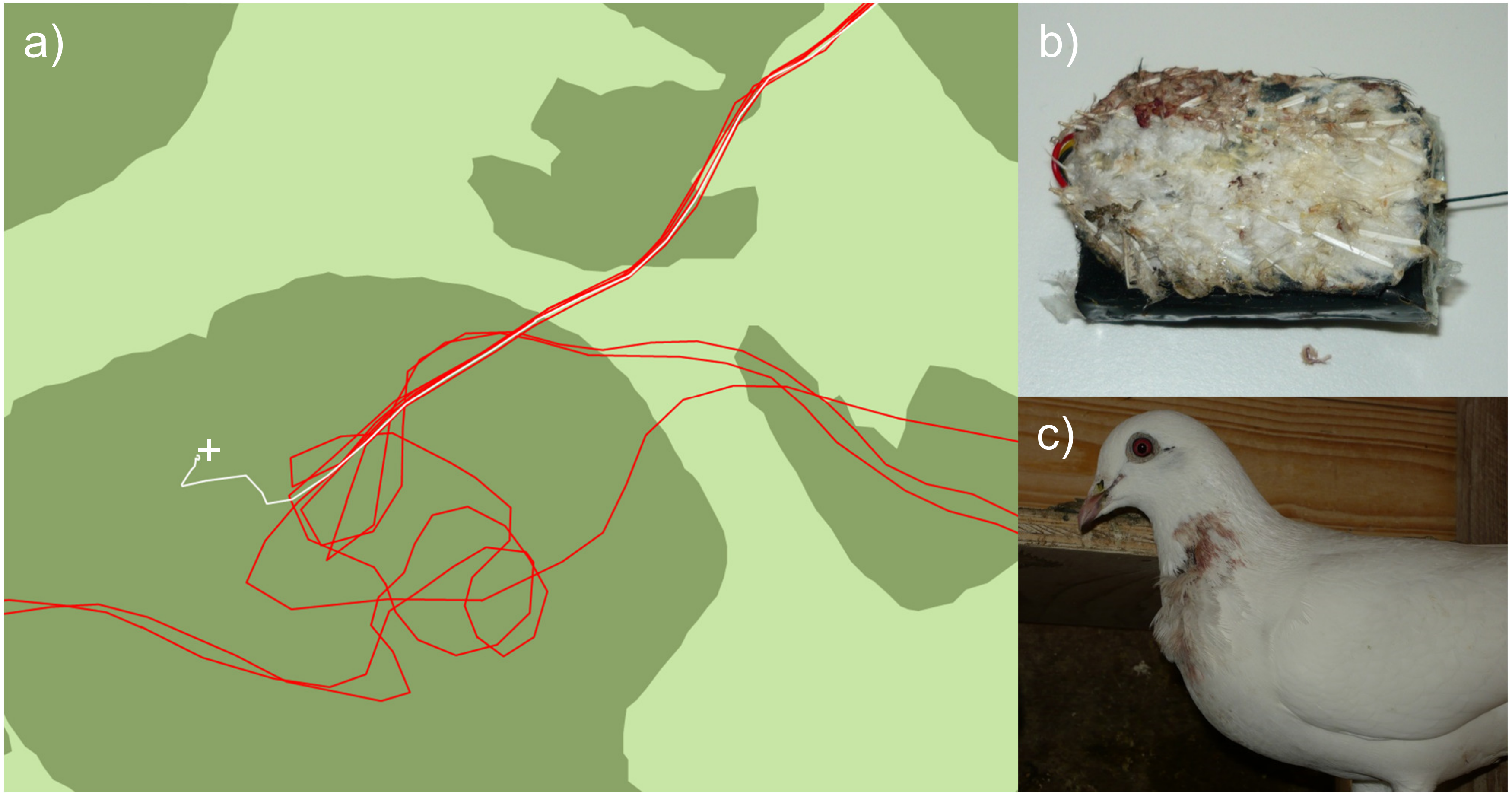

Supplement: Supplementary Information [file srep15490-s1.pdf]
